# The Autocratic Matron

**Published:** 1909-02-20

**Authors:** 


### Nursing Administration.

### THE AUTOCRATIC MATRON.

Strictly speaking the word autocratic is the last which can properly be applied to matrons. The autocrat, if regard is to be paid to derivations, is one whose power is self conferred, and subject to no restraints. The matron's power, on the contrary, is derived from others, and is exercised within strict limits. Yet the quality generally recognised as "autocratic" attaches to matrons in common repute, and it is interesting to trace how this has come about. The matron's authority in a hospital is a far more evident fact than the authority of, for example, the head of a college or of a factory. It expects and requires a degree of obedience which nurses often find irksome, and which outsiders consider ridiculous. The main origin of this authority, which has not come into being accidentally, is the overwhelming importance of the things which pertain to the matron's sphere. Many people whose households are thrown into the wildest confusion if two or three of its inmates are confined to their beds with mild attacks of influenza, seem to believe that hundreds of patients, suffering from differing complaints in acute stages can be attended to automatically, by merely collecting a sufficient number of nurses. It does not occur to them that the energies and wits of every person in the hospital need to be kept perpetually on the alert to prevent any one of these sufferers from being neglected, and to promote the recovery of all. The matron, who is responsible for the care of all these helpless individuals, is driven by the greatness of the charge to that disposition of insistent watchfulness, wrongly stigmatised as autocratic. It matters so much, so terribly, whether her orders are strictly obeyed, that from sheer anxiety she sometimes gets into the habit of adopting a peremptoriness which irks her subordinates. "She takes pleasure in finding fault," it will be said of some capable administrators. It may be so. It is her duty, if fault there be, to discover it. Her greatest difficulties have arisen from faults undiscovered, which she blames herself for not having discovered. It is natural, therefore, that she should be thankful when, faults having been committed, she has been sufficiently vigilant to find them. The peremptory manner is, however, often overdone, and it may generally be regarded in a capable matron as one of "the defects of her qualities."

The autocratic manner is not confined to capable matrons. It is, alas, a common feature of the incapables in whatever position they are found. It has the air of ignoring serious abuses, accompanies acts of favouritism and injustice, and emphasises endlessly its owner's sense of her own dignity. It is discoverable in the youngest probationer, and nothing has a better effect in eradicating it than the training afforded by a first-rate hospital. It is always most objectionable in the head of an ill-managed household; but it is, perhaps, not more harmful to the comfort of all concerned than the easy good nature of the woman who does not realise the importance of her work, and a little self-reliant autocracy sometimes comes as a relief to those who have had to obey the orders of a weak woman, or, rather, who have had to find out without being told what they imagine she would like them to do. The all-important matter in hospital work is the performance of the work. If this is neglected, if the comfort of every member of the household is disregarded, if patients and staff are alike ill cared for, it is not the friendly manners of the matron which can save the situation.

In justice to many worried matrons it must be remembered that the autocratic manner in its worst sense—the manner which seems to ignore the fatigues of the staff, the hardships of over-work, and the discomforts of an ill-mounted establishment—is sometimes due not to incapacity, but to stoicism. The head of the household can do much, but she cannot make bricks without straw. It sometimes happens that able women accept posts in institutions where the most ordinary needs of hardworking women are left unsupplied. They have to rule a household where, to use the old expressive phrase, "Want must be their master." Under these circumstances they gradually surrender to the evil conditions around them, and come in time to identify themselves with the power which inflicts privations pressing more hardly on them than on their subordinates. Most of the complaints which find their way into the public Press against matrons are due to the reticence with which matrons will bear to hear themselves condemned for matters in which they are merely reluctant ministers. We believe this to be a mistaken attitude. In so far as it is dictated by loyalty it is good; but too often it is merely the outcome of an exaggerated officialism. The matron fears by speaking to make mischief. She hides her own dissatisfaction under a veil of stern unconcern to the murmurs around her, and frequently suffers intensely from the unpopularity this entails. We believe that in numerous cases where the matron's reticence is misunderstood a few sensible words would put her in a better position with the staff, and enable her fellow-workers to bear the burden with a more cheerful heart. The best remedy against a manner overcharged with authority is for the matron to cultivate a mutual sense of confidence towards those, like her ward sisters, to whom she must delegate her authority. To explain every rule and apologise for every bit of extra work is not required. But much will be borne if it can be made clear that it is the exigencies of the work, and not individual caprice, which are at the bottom of an unsatisfactory state of affairs, and the wise matron will not disdain sometimes to expose the springs of her actions to her staff.

There are few positions in life which bring with them a more over-powering sense of responsibility and isolation than that of matron. Every hour her insufficiency is brought forcibly before her in the control of those weighty matters pertaining to her sphere. Does the life tend towards a habit of autocracy? Speaking from intimate knowledge of many great matrons, we should say rather it opened a path to rare self-knowledge and humility.

